# Incidence and Risk Factors of Postcontrast Acute Kidney Injury in Patients with Acute Ischemic Stroke

**DOI:** 10.1155/2020/7182826

**Published:** 2020-04-01

**Authors:** Sirichai Chusiri, Aurauma Chutinet, Nijasri Charnnarong Suwanwela, Chankit Puttilerpong

**Affiliations:** ^1^Department of Pharmacy Practice, Faculty of Pharmaceutical Sciences, Chulalongkorn University, Bangkok, Thailand; ^2^Division of Neurology, Department of Medicine, Faculty of Medicine, Chulalongkorn University, Bangkok, Thailand; ^3^Chulalongkorn Stroke Center, Chula Neuroscience Center, King Chulalongkorn Memorial Hospital, Thai Red Cross Society, Bangkok, Thailand

## Abstract

**Background:**

Multimodal computed tomography (CT) guides decision-making regarding use of thrombolytic agents in acute ischemic stroke patients. However, postcontrast acute kidney injury (PC-AKI) is a potential adverse effect of the contrast media used, which may require hemodialysis and cause a longer hospital stay. The incidence and risk factors of PC-AKI in acute ischemic stroke patients, particularly in Thailand, remain unclear. *Goal*. We aimed at determining the incidence and risk factors of PC-AKI in patients with acute ischemic stroke undergoing multimodal CT.

**Methods:**

We conducted a retrospective review of Thai acute ischemic stroke patients admitted to the King Chulalongkorn Memorial Hospital between January 2014 and December 2017 who received multimodal CT and thrombolytic treatment with alteplase.

**Result:**

Overall, 109 patients were included for analysis; eight patients (7.3%) developed PC-AKI. Estimated glomerular filtration rate (eGFR) ≤ 30 mL/min and mechanical thrombectomy were risk factors significantly associated with PC-AKI.

**Conclusion:**

The incidence of PC-AKI in a real practice setting did not differ from previous reports. Two factors were associated with PC-AKI, eGFR ≤ 30 mL/min and mechanical thrombectomy. Patients without these risk factors may not need to wait for the results of renal function testing prior to multimodal CT.

## 1. Introduction

Stroke is the second leading cause of mortality worldwide and the leading cause of morbidity in the elderly. In 2018, approximately 6.45 million people died from stroke; 40% of these deaths occurred in developing countries [1, 2]. Stroke impacts not only affected patients but also their families, who provide care for them at home. The estimated lifetime cost of stroke care is estimated to range between 59,800 and 230,000 USD per patient [3]. There are 2 types of stroke, ischemic and hemorrhagic, and each has different treatment. Prompt and accurate diagnosis of ischemic stroke is crucial for appropriate management with administration of thrombolytic drug therapy, which can restore cerebral blood flow and prevent morbidity and mortality [4].

Multimodal computed tomography is the recommended imaging investigation in acute ischemic stroke. It is faster and less costly than magnetic resonance imaging [5] and consists of noncontrast computed tomography (NCCT), computed tomography angiography (CTA), and computed tomography perfusion (CTP). NCCT is first used to differentiate between hemorrhagic and ischemic stroke. Then, CTA and CTP evaluate cerebral ischemia and arterial occlusion to guide treatment decision-making regarding thrombolytic drug administration or mechanical thrombolysis procedures. A potential adverse effect of CTA and CTP is postcontrast acute kidney injury (PC-AKI), which may result in prolonged hospitalization and require additional treatment, including hemodialysis [6]. Therefore, kidney function is evaluated prior to multimodal CT. However, this may cause a delay in treatment, which could seriously affect disease prognosis. A wide range of PC-AKI incidence rates has been reported in ischemic stroke patients. This might be due to variable criteria and definitions of PC-AKI among the studies as well as different study designs. In Europe and the US, the incidence of PC-AKI after CTA or CTP reportedly ranges from 1.5% to 14.8% [7–12]. The risk of PC-AKI may be elevated in patients with older age, long-term and uncontrolled diabetes, renal insufficiency, and concurrent use of nephrotoxic drugs [13]. Therefore, we aimed at identifying risk factors for PC-AKI among these patients to help guide proper management.

## 2. Materials and Methods

This study was approved by the Institutional Review Board of the Faculty of Medicine at Chulalongkorn University. We retrospectively reviewed the medical records of Thai acute ischemic stroke patients who were admitted to the King Chulalongkorn Memorial Hospital between January 2014 and December 2017. Patients without serum creatinine data within 7 days after receiving contrast media were excluded. The primary outcome was incidence of PC-AKI. Secondary outcomes were hemodialysis requirement, length of hospital stay, and mortality.

According to the stroke protocol of our hospital, patients with suspected acute ischemic stroke receive clinical assessment, intravenous access, and blood sample collection prior to multimodal CT. Imaging studies are obtained without waiting for laboratory results. Blood samples are evaluated for various laboratory parameters including serum urea nitrogen, serum creatinine, and estimated glomerular filtration rate (eGFR) before and within 72 hours after multimodal CT. The eGFR is calculated by the Chronic Kidney Disease Epidemiology Collaboration (CKD-EPI) equation. The first patient's serum creatinine was defined as the baseline serum creatinine. All patients in this study were given standard dose alteplase and evaluated for hemorrhagic transformation with NCCT 24 h later. The diagnosis of PC-AKI was defined as a >0.3 mg/dL increase in serum creatinine or ≥1.5–1.9 times increase from baseline within 72 hours [14].

Demographic data, vital signs, laboratory data, total fluid volume administered in the first 24 and 48 h, past medical history (hypertension, anemia, diabetes mellitus, chronic kidney disease (eGFR baseline ≤ 60 mL/min), atrial fibrillation, dyslipidemia, myocardial infarction, congestive heart failure, and current use of metformin), and ischemic stroke complications in the first 7 days (hemorrhagic infarction type I (HI-1), hemorrhagic infarction type II (HI-II), parenchymal hematoma type I (PH1), and parenchymal hematoma type II (PH2)), brain edema, urinary tract infection, and progressive stroke) were recorded. In addition, stroke-relevant data, such as signs and symptoms, time from admission to treatment, the National Institute of Health Stroke Scale (NIHSS) score, and the modified Rankin scale score were recorded. The contrast media used in this study was iopromide (Ultravist®, Bayer HealthCare LLC, Whippany, NJ, USA). Each patient received 50 mL of contrast media for brain CTA and 30 mL for neck CTA. For CTP, each patient received 50 mL of contrast media via intra-arterial injection. Therefore, the total amount of contrast media per patient did not exceed 130 mL.

### 2.1. Statistical Analysis

Statistical analysis was performed using the SPSS software, version 22 (IBM Corp., Armonk, NY, USA). Quantitative data are reported as means and standard deviation; qualitative data are shown as numbers and percentage. Incidence was defined as the number of cases divided by the total number of patients included in the study during the specified time period. To determine risk factors associated with PC-AKI, bivariate analyses and multivariate logistic regression were conducted. All variables with a *P* value of < 0.20 in the bivariate analysis were included in the initial logistic regression model. Variables were then removed in a stepwise fashion from the model based on the highest *P* value. Goodness of fit for each stepwise model was compared with the likelihood-ratio test. A *P* value of < 0.05 was considered significant.

## 3. Results

During the study period, 191 stroke patients received contrast media for multimodal CT. Eighty-two patients were excluded due to lack of serum creatinine data within 7 days after imaging. Finally, 109 patients (57%) were included for analysis.

Fifty-nine patients (54.1%) were female. The average patient age was 67.38 ± 14.13 years. PC-AKI occurred in 8 patients (7.3%). Characteristics of the control group (patients without PC-AKI) and the case group (patients with PC-AKI) are shown in Table 1. Significant differences between the control and case groups were found with respect to baseline hematocrit (39.56 ± 5.39 vs. 33.60 ± 7.00, *P* = 0.013) and baseline hemoglobin (13.12 ± 1.85 vs. 11.10 ± 2.71 g/dL, *P* = 0.015). Other characteristics, including the NIHSS and modified Rankin scores, stroke complications, and volume of contrast media administered did not differ between the groups. The average time from admission to alteplase administration was 52.3 minutes. Six patients (5.5%) received mechanical thrombectomy.

Eight patients developed PC-AKI. The characteristics of each are shown in Table 2. Seven were graded as severe acute renal failure and only 1 required hemodialysis. Fifteen patients died overall; 6 of these were patients who developed PC-AKI (3 died from hemorrhagic transformation, 2 died from infection, and 1 died from large infarction). Although the average length of hospital stay was longer for patients with PC-AKI than those without PC-AKI, the difference was not significant (22 d vs. 16 d, *P* = 0.309).

For the initial logistic regression model, the following variables were included: (1) age 60–75 years, (2) SBP of ≥160 mmHg on admission, (3) total fluids administered within 24 hours of admission, (4) baseline eGFR of ≤30 mL/min, (5) anemia, and (6) mechanical thrombectomy. Though SBP on admission, baseline serum creatinine, baseline hemoglobin, baseline hematocrit, and hypertension had *P* values of < 0.20, we did not include them in the model, but those that overlap with the above variables were selected. After stepwise removal of variables, two significant variables for the development of PC-AKI were found: mechanical thrombectomy and eGFR 30 mL/min (Table 3).

## 4. Discussion

This is the first study to examine PC-AKI in acute stroke patients in Thailand who underwent multimodal CT and received alteplase. All patients underwent imaging without waiting for serum creatinine results, which differs from previous case-control studies. In general, PC-AKI describes a decrease in renal function that follows intravascular administration of contrast media. The decrease in renal function is usually mild and peaks 2–3 days after contrast is administered; therefore, we used the 2018 European Society of Urogenital Radiology criteria to define PC-AKI [14].

A previous study reported a 2.6% incidence of PC-AKI after multimodal CT in acute stroke [10]; however, our rate was higher (7.3%). We excluded 82 (43%) of the eligible patients from analysis because they did not have kidney function testing within 72 hours after receiving contrast media. However, data from these excluded patients showed no evidence of renal dysfunction 7–14 days after contrast administration, so we assume that no patient in this group developed PC-AKI. If we recalculate the incidence of PC-AKI by including them, the incidence of PC-AKI in this study was 4.2%, similar to the previous one. Most of the patients with PC-AKI (7 of 8) developed severe renal failure which required hemodialysis in one. Severe renal failure may be the result of direct kidney damage, not a temporary change in kidney function during hospital admission [15]. However, the length of hospital stay did not significantly differ between patients who developed PC-AKI and those who did not.

We did not include the variables of hemorrhagic transformation in the analysis for risk factors for developing PC-AKI because hemorrhagic transformation was assessed 24 h after receiving alteplase. Patients may develop PC-AKI before hemorrhage is evaluated; therefore, it is difficult to conclude that it is a true risk factor for PC-AKI. In addition, impaired renal function might be a risk factor for cerebral hemorrhage because renal insufficiency causes platelet dysfunction and induces platelet-vessel wall interaction [16].

Logistic regression analysis found two significant risk factors for developing PC-AKI. One was eGFR of ≤30 mL/min that is a known risk factor for PC-AKI in patients receiving low osmolar contrast media (LOCM), the same type of contrast media used in this study [15]. However, only three of our patients had eGFR of ≤30 mL/min; the number of patients may be too small to definitively conclude that it is a risk factor for PC-AKI. In addition, this group of patients is often excluded from general PC-AKI studies. The second risk factor was mechanical thrombectomy. In patients receiving thrombectomy, an additional 50 mL of contrast media was needed for intra-arterial injection. The total contrast media volume received was therefore 180 mL in these patients. Previous studies have noted that contrast media doses of 30–125 mL have a relatively low risk of kidney toxicity [17, 18]; however, higher doses increase the risk. Another consideration is that intra-arterial injection of contrast media may have a higher risk of causing PC-AKI than intravenous injection, but the evidence is conflicting [19, 20]. In this study, only six patients received thrombectomy; the number of patients is too small to definitively consider it as a risk factor for PC-AKI. Future studies with a larger number of patients are required. Nonetheless, this study demonstrates that multimodal CT treatment is safe and associated with low PC-AKI incidence. There is no need to delay imaging and alteplase treatment in ischemic stroke patients while waiting for kidney function testing results, as delay could result in worse patient outcome.

Limitations of this study include its retrospective design, incomplete clinical data in a considerable proportion of otherwise eligible patients, and small sample size. Further prospective studies are needed to more precisely determine PC-AKI risk factors in acute stroke patients and develop guidelines.

## 5. Conclusions

Multimodal CT during acute stroke evaluation is safe with respect to renal function and does not delay evaluation or appropriate treatment. The incidence of PC-AKI in this real practice setting was not significantly different from previous reports. Baseline renal function is an important marker to identify patients at risk. Patients with elevated serum creatinine and those undergoing thrombectomy require intensive monitoring to prevent PC-AKI. Further studies to validate PC-AKI risk factors are warranted to increase the safety of contrast media use in acute stroke patients.

## Figures and Tables

**Table 1:** Patient characteristics.

Baseline characteristic	Number of patients (%)		*P* value
	Control (*n* = 101)	Case (*n* = 8)	
Age (years), mean ± SD	67.30 ± 14.57	68.38 ± 6.99	0.835
(i) <60	29 (28.7)	0 (0)	—
(ii) 60–75	39 (38.6)	7 (87.5)	0.027^∗^
(iii) >75	33 (32.7)	1 (12.5)	0.262
Sex			
(i) Male	48 (47.5)	2 (25)	0.234
Systolic blood pressure (SBP), mean ± SD	155.01 ± 29.15	173.00 ± 30.97	0.106
(i) SBP ≥ 160 mmHg	38 (37.6)	5 (62.5)	0.180
Body weight (kg), mean ± SD	61.31 ± 13.24	59.23 ± 9.54	0.662
Patient volume status			
Total fluids within 24 h (mL), mean ± SD	2173 ± 846	2723 ± 1937	0.129
Total fluids within 48 h (mL), mean ± SD	4564 ± 1542	5281 ± 2484	0.230
Laboratory data			
Baseline serum creatinine (mg/dL), mean ± SD	1.09 ± 0.56	1.79 ± 2.47	0.078
(i) ≥1.5	9 (8.9)	2 (25)	0.167
Baseline eGFR (mL/min), mean ± SD	70.81 ± 25.09	61.92 ± 23.47	0.323
(i) ≤30	2 (1.98)	1 (12.5)	0.128
Baseline hematocrit, mean ± SD	39.56 ± 5.39	33.60 ± 7.00	0.013^∗^
Baseline hemoglobin (g/dL), mean ± SD	13.12 ± 1.85	11.10 ± 2.71	0.015^∗^
Stroke severity score			
NIHSS on admission, mean ± SD	13.85 ± 6.83	12.38 ± 7.71	0.559
Modified Rankin scale on admission, mean ± SD	4.04 ± 1.25	4.00 ± 1.23	0.940
Past medical history			
Diabetic mellitus	25 (24.8)	1 (12.5)	0.446
Chronic kidney disease	36 (35.6)	3 (37.5)	0.916
DM and chronic kidney disease	14 (13.9)	1 (12.5)	0.914
Hypertension	63 (62.4)	7 (87.5)	0.186
Congestive heart failure	6 (5.9)	0 (0)	—
Anemia	32 (31.7)	4 (50)	0.202
Myocardial infarction	12 (11.9)	1 (12.5)	0.416
Dyslipidemia	43 (42.6)	3 (37.5)	0.780
Atrial fibrillation	25 (24.8)	2 (25)	0.988
Current metformin use	9 (8.9)	0 (0)	—
Stroke complication			
Brain edema	9 (8.9)	1 (12.5)	0.736
Progressive stroke	6 (5.9)	0 (0)	—
Urinary tract infection	11 (10.9)	2 (25)	0.350
Procedure and volume of contrast administered			
Volume of contrast media, mean ± SD	127.82 ± 16.47	130 ± 0	0.705
Volume/body weight ratio (V/B), mean ± SD	2.21 ± 0.52	2.24 ± 0.33	0.857
Mechanical thrombectomy	4 (3.9)	2 (25)	0.030^∗^

^∗^ *P* < 0.05.

**Table 2:** Characteristics of the eight patients with PC-AKI.

Pt.	Age (year)	NIHSS score	SBP (mmHg)	Initial serum Cr (mg/dL)	Initial eGFR (mL/min)	Contrast volume (mL)	Hemorrhagic transformation	Symptom PC-AKI	Hemodialysis	Death
1	75	19	155	0.55	92.20	180	—	Severe acute renal failure	—	✓
2	62	2	160	0.80	71.92	180	—	Severe acute renal failure	—	✓
3	70	17	127	7.86	13.92	130	—	Severe acute renal failure	✓	—
4	67	22	187	0.9	63.28	130	—	Severe acute renal failure	—	✓
5	62	13	183	0.83	69.70	130	PH2	Severe acute renal failure	—	✓
6	80	6	197	1.50	56.40	130	PH2	Severe acute renal failure	—	✓
7	60	3	150	0.73	78.66	180	PH2	No symptom	—	—
8	71	17	225	1.18	49.24	130	PH2	Severe acute renal failure	—	✓

**Table 3:** Variables associated with development of PC-AKI.

Variables	*B*	Adjusted OR	95% CI	*P* value
Age 60–75 years	2.464	11.749	1.049–131.622	0.056
Total fluids within 24 hours	0.000	1.000	0.999–1.001	0.924
Mechanical thrombectomy	3.530	34.127	1.383–842.022	0.031^∗^
Anemia	0.738	2.092	0.250–17.489	0.496
SBP ≥ 160 mmHg on admission	2.007	7.438	0.706–78.380	0.095
eGFR ≤ 30 mL/min	4.149	63.363	0.980–4097.673	0.048^∗^

^∗^ *P* < 0.05.

## Data Availability

The patient's data used to support the findings of this study were supplied by the Ethics Committee of Chulalongkorn Hospital (IRB No.665/60) under license and so cannot be made freely available. Requests for access to these data should be made to Sirichai Chusiri, sirichai.chusiri@gmail.com.
